# Big fish, little divergence: phylogeography of Lake Tanganyika’s giant cichlid, *Boulengerochromis microlepis*

**DOI:** 10.1007/s10750-014-1863-z

**Published:** 2015-04-01

**Authors:** Stephan Koblmüller, Elizabeth A. Odhiambo, Danny Sinyinza, Christian Sturmbauer, Kristina M. Sefc

**Affiliations:** Department of Zoology, Karl-Franzens-University Graz, Universitätsplatz 2, 8010 Graz, Austria. stephan.koblmueller@uni-graz.at; Department of Zoology, Karl-Franzens-University Graz, Universitätsplatz 2, 8010 Graz, Austria; Ichthyology Section, National Museums of Kenya, Nairobi, Kenya; Department of Fisheries, Ministry of Agriculture and Lifestock, Mpulungu, Zambia; Department of Zoology, Karl-Franzens-University Graz, Universitätsplatz 2, 8010 Graz, Austria; Department of Zoology, Karl-Franzens-University Graz, Universitätsplatz 2, 8010 Graz, Austria

**Keywords:** Boulengerochromini, Cichlidae, Demography, Genetic diversity, Mitochondrial DNA

## Abstract

The largely endemic cichlid species flocks of the East African Great Lakes are among the prime examples for explosive speciation and adaptive radiation. Speciation rates differ among cichlid lineages, and the propensity to radiate has been linked to intrinsic and extrinsic factors such as sexual selection and ecological opportunity. Remarkably, only one cichlid tribe—the Boulengerochromini—comprises just a single species, *Boulengerochromis microlepis*, a predominantly piscivorous endemic of Lake Tanganyika and the world’s largest cichlid. While the lineage diverged from its closest relatives at the onset of the Lake Tanganyika radiation >8 MYA, mitochondrial control region sequences collected in this study dated the most recent common ancestor of *B. microlepis* to ~60–110 KYA. There was no evidence of phylogeographic structure in the lake-wide sample. Patterns of genetic diversity and demographic analyses were consistent with slow and steady population growth throughout the reconstructed timescale. Additionally, the shallow divergence within the species may be related to a possibly large variance in reproductive success in this highly fecund species. Trophic niche space restriction by sympatric piscivores, lack of geographic structure, low potential for sexual selection arising from the monogamous mating system and extinction may have contributed to keeping the lineage monotypic.

## Introduction

The largely endemic cichlid species flocks of the East African Great Lakes represent one of the most outstanding examples of explosive speciation and adaptive radiation (Fryer & Iles, 1972; Salzburger,2009; Sturmbauer et al., 2011). It is one characteristic of adaptive radiation that not all lineages that seeded these radiations actually diversified and if they did, to a similar extent. Some lineages are species-rich whereas others have produced only a few species. Such differences in species richness among subgroups of an endemic species assemblage have been linked to a different potential to radiate due to preadaptive phenomena such as the possession of particular lineage-specific key innovations (e.g., Mayr, 1963; Liem, 1973; Sanderson & Donoghue, 1994). Thus, the particular anatomy of the pharyngeal apophysis providing a second set of jaws and a highly specialized reproductive behavior (Liem, 1973; Crapon de Crapona, 1986) have been proposed as key innovations underlying the evolutionary success of cichlids as compared to other sympatric fish lineages. However, not all cichlid lineages that colonized emerging lakes diversified to a similar extent. Rather, some lineages are species-rich whereas others have produced a few or even a single species, despite similar age. These differences in species richness have been attributed to differences in ecological characteristics, in particular habitat preferences of the different cichlid lineages. In Lake Malawi, for example, highly stenotopic rock-dwelling cichlid species tend to be philopatric and gene flow between geographically close populations separated by habitat discontinuities in the form of sandy stretches of shoreline, river estuaries, or deep water is low (e.g., van Oppen et al., 1997; Markert et al., 1999; Rico & Turner, 2002; Pereyra et al., 2004). Cichlids occupying shallow sandy habitats show less population structure (Taylor & Verheyen, 2001; Pereyra et al., 2004; Anseeuw et al., 2011), and benthopelagic species show hardly any or no significant genetic structure at all on a lake-wide scale (Shaw et al., 2000; Genner et al., 2008, 2010b). For Lake Tanganyika, thus far, population genetic data are only available for rock-dwelling cichlid species and taxa preferring the intermediate habitat at the rock–sand interface in shallow water. Nevertheless, similar trends have been observed in Lake Tanganyika: Highly stenotopic rock-dwellers show high levels of geographic structuring, whereas less stenotopic taxa and taxa inhabiting the intermediate habitat are less structured (Meyer et al., 1996; Taylor et al., 2001; Duftner et al., 2006; Koblmüller et al., 2007, 2009,2011; Koch et al., 2007; Sefc et al., 2007; Takahashi et al., 2009; Wagner & McCune, 2009; Nevado et al.,2009, 2013; Van Steenberge et al., 2014). The overall pattern suggests that dispersal capacity is modulated by adaptation to particular habitat types and ecological niches during the radiation process (Sturmbauer,1998). Assuming that geographic population structuring is an important prerequisite for allopatric speciation (Turelli et al., 2001), lineages composed of stenotopic rock-dwellers should be particularly species-rich, whereas lineages comprising mobile benthopelagic taxa should be comparatively species poor. Indeed, in each of the three East African Great Lakes, Tanganyika, Malawi, and Victoria, patterns of cichlid diversity appear to follow this model (Turner, 1996; Seehausen et al., 1997; Koblmüller et al., 2008). Nonetheless, that a particular lineage did not diversify at all, is quite unusual. This is the case for the endemic Lake Tanganyika cichlid tribe Boulengerochromini, which only comprises the species *Boulengerochromis microlepis*, one of the few benthopelagic top-predators of the lake.

Previous studies have shown that environmental influences, mainly climatically driven lake level fluctuations (Cohen et al., 1997; Scholz et al., 2007; McGlue et al., 2008), have synchronized population divergence and patterns of past population size changes of stenotopic rock-dwelling cichlids within and across Lake Tanganyika and Malawi (Sturmbauer et al., 2001; Genner et al., 2010a; Koblmüller et al.,2011; Nevado et al., 2013). Whether population histories of highly mobile benthopelagic or truly pelagic species have been equally affected by large lake level fluctuations remains largely unknown.

Lake Tanganyika’s giant cichlid, *B. microlepis*, the only member of the tribe Boulengerochromini (Takahashi, 2003), is distantly related to all other cichlid species of this lake, so that it is assumed that its ancestor colonized the lake as one of the seeding lineages of the radiation (Salzburger et al., 2002). The phylogenetic relationships of *B. microlepis* are still tentative and a matter of discussion. Molecular phylogenetic analyses based on mtDNA (Klett & Meyer, 2002; Koblmüller et al., 2005) placed *B. microlepis* as closely related to the tribe Tilapiini (sensu Dunz & Schliewen, 2013), which does not occur in the lake itself but has a widespread distribution from the Congo basin and Lake Malawi southwards to South Africa. Nuclear data, on the other hand, imply that *B. microlepis* is part of the lacustrine assemblage, representing either its most basal lineage or being most closely related to the mainly bathypelagic genera *Bathybates, Hemibates*, and *Trematocara* (Nishida, 1997; Muschick et al., 2012; Dunz & Schliewen, 2013; Friedman et al., 2013). However, independent of the genetic marker used to infer phylogenetic relationships and the approaches employed to estimate divergence times, the Boulengerochromini are an old lineage that diverged from its closest relatives at least ~8 MYA (Genner et al.,2007; Koblmüller et al., 2008; Schwarzer et al., 2009; Friedman et al., 2013). With a total length exceeding 80 cm (Matthes, 1961; personal observation by SK), *B. microlepis* is considered the world’s largest cichlid fish species (Coulter, 1991). It is found over a rather wide depth range from the very shallow water down to a depth of 150 m in all kinds of habitat—though only infrequently over rocky substrate—preying mainly upon various fish species, but also on crabs, shrimps, molluscs, and insect larvae (Kawabata & Mihigo,1982; Coulter, 1991; Bayona, 1991a; Sturmbauer et al., 2008). *B. microlepis* is a substrate breeding, probably semelparous species, breeding in rather shallow sandy or intermediate habitat, with both male and female guarding their numerous (up to 12,000) fry (Poll, 1956; Kuwamura, 1986; Konings, 1998; Büscher, 2009). Occasionally, large numbers of breeding pairs can be observed in very shallow water (personal observation by SK). As a highly valued and pricy staple fish, *B. microlepis* is heavily targeted by local fishermen. One of its popular local names, “English fish,” reflects its popularity among well-to-do, typically English-speaking, people. Previous catch statistics for beach-seining indicate that the species used to be very abundant and constituted a remarkably high portion of the total yield (Bayona, 1991b). As beach-seining has been prohibited over most parts of Lake Tanganyika, the species is nowadays mainly caught by hook and line, often in very deep water (personal observation by SK). Currently, it does not seem to be threatened by overfishing as catch statistics for the very southern part do not indicate a population decline over the last decade (Sinyinza, unpublished data).

To date there have been no studies of spatial or temporal population genetic structure of *B. microlepis* or any other large predatory and highly mobile Lake Tanganyika cichlid fish species. Such studies may not only increase our understanding about factors and processes shaping intraspecific diversity, but might also help to identify potentially segregated fish stocks and thus provide important insights for conservation and fisheries management. Here, we characterize the genetic diversity of *B. microlepis* and reconstruct its phylogeographic structure and past population size trajectories based on DNA sequences of the most variable part of the mitochondrial control region. The findings are discussed in the light of the hydrologic history of Lake Tanganyika and the biological characteristics of the species.

## Materials and methods

**Fig. 1:**
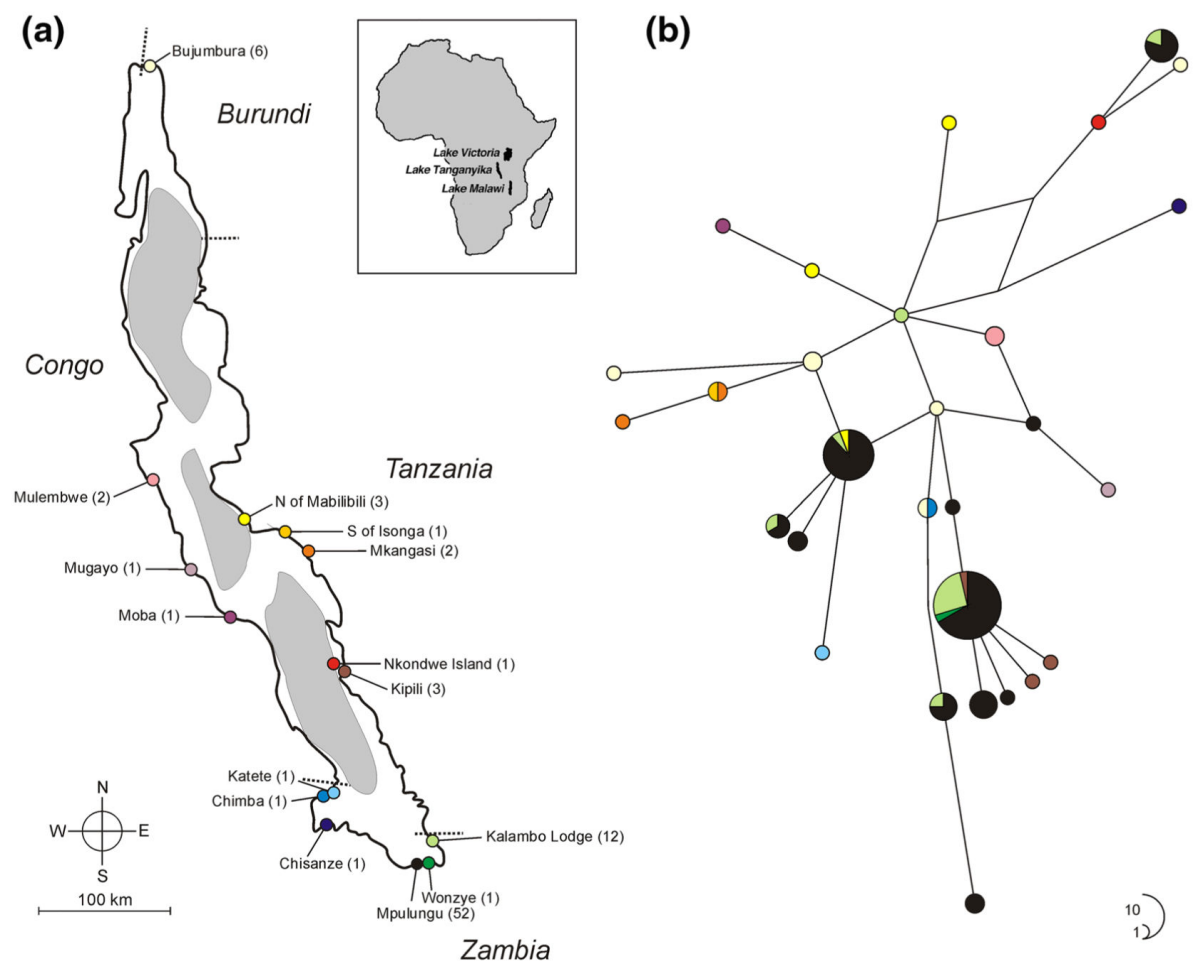
**a** Map of Lake Tanganyika showing the sampling localities. *Numbers in parentheses* refer to sample sizes. The three deep-water basins are indicated by *gray shading*. **b** Median-joining (MJ) network of *B. microlepis* haplotypes. *Circle* sizes are proportional to haplotype frequency and connecting *lines* are proportional to mutation events between haplotypes. *Different colors* refer to different sampling localities as shown in **a**

Fin clips were taken from 88 individuals of *B. microlepis* collected with gill net or harpoon from 13 localities in Lake Tanganyika, or obtained at local fish markets in Bujumbura and Mpulungu or from artisanal fishermen on the lake, during several field trips between 2001 and 2013 (Fig. 1; Supplementary Table 1), and preserved in 96% ethanol. Whole genomic DNA was extracted following a standard Chelex protocol (Walsh et al., 1991). The most variable part of the mitochondrial control region was amplified and sequenced according to the protocols described in Koblmüller et al. (2011) and Duftner et al. (2005), respectively. The primers used for PCR and chain termination sequencing were L-Pro-F_Tropheus (Koblmüller et al., 2011) and TDK-D (Lee et al.,1995). DNA fragments were purified with Sephadex™ G-50 (Amersham Biosciences) and visualized on an ABI 3130×l capillary sequencer (Applied Biosystems). Sequences were aligned by eye in Mega 5.1 (Tamura et al., 2011). The length of the final alignment (including gaps) was 358 bp. Sequences are deposited in GenBank under the accession numbers KJ438258–KJ438286.

Phylogenetic relationships among haplotypes were visualized by a full median-joining (MJ) network (Bandelt et al., 1999), by setting the weighted genetic distance parameter ε to 90 [10 (default character weight) × 9 (the observed maximum number of pairwise differences)] and activating the “MJ square” option, followed by maximum parsimony post-processing (Polzin & Daneschmand, 2003) as implemented in Network (version 4.6; available at www.fluxus-engineering.com/sharenet.htm). Haplotype (Hd) and nucleotide diversity (p) were calculated in DnaSP 5.10 (Librado & Rozas, 2009). Spatial genetic structure of *B. microlepis* was analyzed by spatial analysis of molecular variance using SAMOVA 1.0 (Dupanloup et al., 2002), which defines groups of populations that are maximally differentiated from each other in a geographically homogeneous environment by maximizing the proportion of genetic variance (*F*_CT_; Wright,1978) among *K* groups. Analyses for *K* = 2–7 were conducted with 100 simulated annealing runs. For each *K*, the configuration with the largest *F*_CT_ values after all 100 independent simulated annealing processes (5,000 iterations) was retained as the best spatially genetic clustering. To test for signals of past population expansion, we calculated a mismatch distribution as well as Fu’s *F*_s_ (Fu, 1997) and Tajima’s *D* (Tajima,1989) in Arlequin 3.5.1.2 (Excoffier & Lischer, 2010). The fit of the observed mismatch distribution to the expectations based on growth parameter estimates was evaluated by the sum of squared differences (*SSD*) and the raggedness index (*rg*). Furthermore, past population size trajectories were inferred by means of a Bayesian coalescent approach [Gaussian Markov random field (GMRF) skyride tree prior; Minin et al., 2008] as implemented in BEAST 1.8.0 (Drummond & Rambaut, 2007). We employed the model of molecular evolution selected by the Bayesian information criterion (BIC) in jModelTest 0.1 (Posada, 2008), assuming a strict molecular clock and a substitution rate of 0.0324 and alternatively 0.057 per site per MY (Genner et al., 2007, 2010a; Koblmüller et al., 2009). Two independent MCMC runs of 1 million generations each were conducted, sampling every 1000th step with a burn-in of the first 10% of sampled generations. Verification of effective sample sizes (ESS > 200 for all parameters), trace of MCMC runs, and visualization of past demographic changes were done in Tracer 1.5 (Rambaut & Drummond, 2009).

## Results

In total 29 haplotypes were detected in 88 individuals. High haplotype (*H*_d_) diversity (*H*_d_ = 0.871) contrasts with low nucleotide diversity (π) and little genetic divergence between haplotypes (π = 0.00951; maximum number of pairwise differences between haplotypes = 9). The MJ network did not indicate geographic structure. Haplotypes are shared between geographically distant localities, sometimes even from opposite ends of the lake (e.g., Bujumbura, Burundi, and Chimba, Zambia) (Fig. 1b). SAMOVA identified four distinct clusters as the grouping with the highest *F*_CT_ (*F*_CT_ = 0.37894, *P* < 0.001) (Supplementary Fig. 1). However, other solutions (with *K* ranging from 2 to 7) achieved similar *F*_CT_ values, and all solutions consisted of one large group spanning a lakewide distribution, and variable numbers of small and geographically restricted groups. Importantly, the scenario of *K* = 1, i.e., one single panmictic population, cannot be tested in the SAMOVA framework. Based on the structure of the haplotype network and the failure of SAMOVA to group individuals geographically, we conclude that *B. microlepis* is connected by high levels of gene flow across Lake Tanganyika. This does not preclude the possibility of population genetic differentiation, which might be detectable with appropriate sampling.

**Fig. 2:**
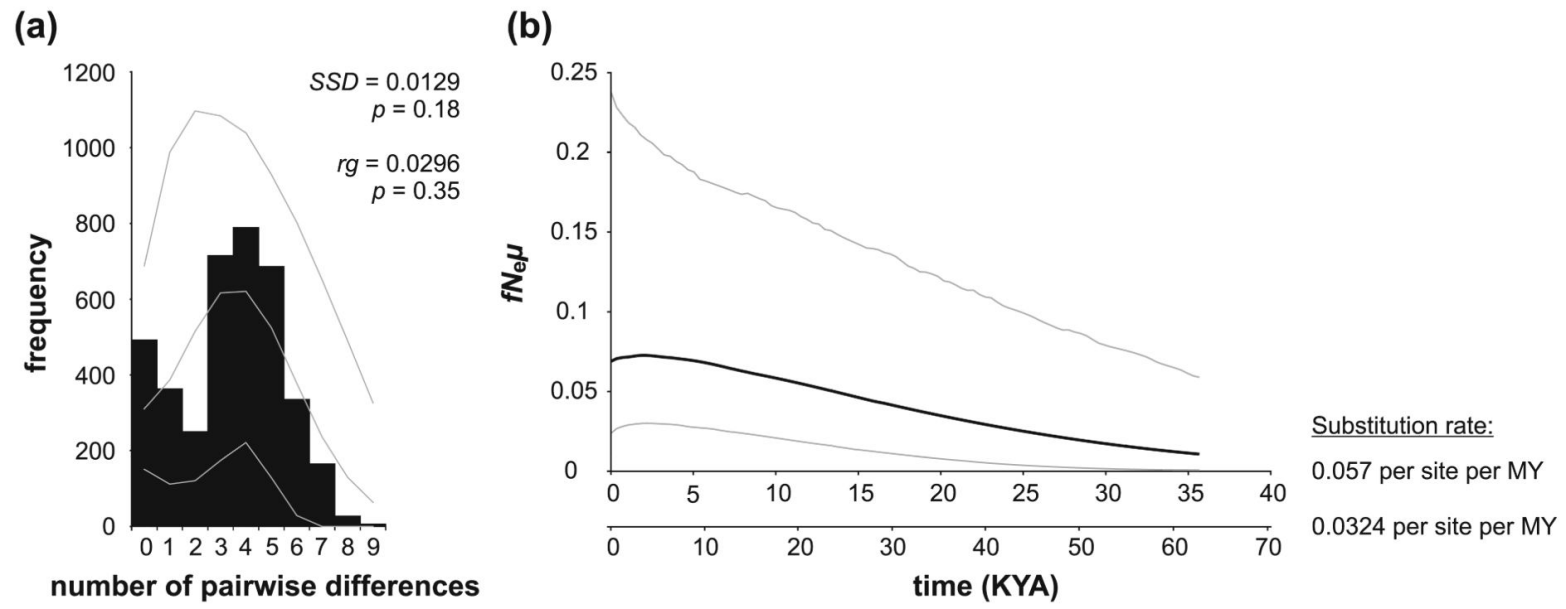
Demographic history of *B. microlepis*. **a** Mismatch distribution. *Black columns* represent the observed frequency of pairwise differences. *Gray lines* refer to the expected distribution based on parameter estimates and their 95% confidence limits simulated under a model of population growth. Sum of squared differences (SSD) and raggedness index (rg) and their respective *P* values are given to describe the fit of the observed mismatch distribution to the expectations based on growth parameter estimates. **b** GMRF skyride plot of past population size trajectories. The skyride plot shows the product of female effective population size (*fN_e_*) and mutation rate (*μ*) through time, assuming substitution rates of 5.7 and 3.24% per site per MY (Genner et al., 2007, 2010a; Koblmüller et al., 2009). The *thick black line* represents the median values; the *thin gray lines* denote 95% highest posterior density (HPD) intervals

The fit of the observed mismatch distribution to the expectations based on growth parameter estimates, with nonsignificant *SSD* and *rg* values (Fig. 2a), and a significantly negative Fu’s *F*_s_ (*F*_s_ = −16.162, *P* < 0.001) are consistent with recent populationgrowth. Tajima’s D was negative, as expected for recent population growth, but not significantly different from zero (*D* = −0.946, *P* = 0.176; but note that the power to detect population expansion is considerably lower for Tajima’s *D* as compared to Fu’s *F*_s_; Ramos-Onsins & Rozas, 2002). Congruently, a GMRF skyride indicated slight continuous population growth over the last approximately 35–60 KY (dependingon the substitution rate assumed) (Fig. 2b). However, the width of the 95% HPD intervals does not reject a scenario with constant population size. The time to the most recent common ancestor (MRCA) was estimated as 63.349–111.447 KYA (respective 95% HPDs: 35.679–94.256, 62.769–165.821), and present female effective population size as 1,203,169–2,110,173(respective 95% HPDs: 413,400–4,166,021; 725,041–7,306,560), both depending on the assumed substitution rate.

## Discussion

Analysis of the most variable region of the mitochondrial control region revealed shallow population divergence and a lack of phylogeographic structure in *B. microlepis*, with several haplotypes shared between individuals from very distant parts of the lake. Mitochondrial divergence within *B. microlepis* lies within the range of divergence found in regional samples of stenotopic littoral cichlids for which the same mitochondrial DNA fragment was analyzed. For example, 2.5% maximum divergence in *B. microlepis* from across the entire lake compare to 2.2-4.2% (mean 3.5%) maximum divergence in Variabilichromis moorii, Eretmodus cyanostictus, Ophthalmotilapia ventralis, Neolamprologus caudopunctatus, and *Perissodus microlepis*, all sampled within southern Zambia (Duftner et al., 2006; Koblmüller et al., 2007, 2009; Sefc et al., 2007). In contrast, maximum divergence in a lake-wide sample of *Neolamprologus pulcher/brichardi* amounted to 8.7% (Duftner et al., 2007). Our sampling is strongly biased toward the very south of Lake Tanganyika, with fewer individuals and locations sampled in the middle and northern parts of the lake. Therefore, tests for population structure suffered from reduced power, and it is possible that deviations from panmixis remained undetected in this study.

*Boulengerochromis microlepis* is one of the toppredators in Lake Tanganyika. As such, *B. microlepis* is highly mobile and not restricted to a particular type of habitat or depth and apparently disperses over long distances without habitat-imposed restrictions. Similar to *B. microlepis*, phylogeographic structure is also lacking in benthopelagic cichlids in the genera *Rhamphochromis* and *Diplotaxodon* from Lake Malawi (Shaw et al., 2000; Genner et al., 2008,2010b). Both genera comprise medium-sized to large offshore predators that feed in the water column, with large fish being almost exclusively piscivorous (Turner et al., 2002). One *Rhamphochromis* species studied in detail, *R. longiceps*, uses lagoons and satellite lakes as nursery areas, which might promote population differentiation if fish remained close to their breeding grounds. Yet, no geographic population structure has been detected, which has been explained by an apparent lack of homing behavior, and dispersal mainly determined by prey availability and opportunistic selection of suitable nursery grounds (Genner et al., 2008). Similarly, four of the eight *Diplotaxodon* species studied so far show no spatial population subdivision, whereas the other four species show slight but significant spatial genetic differences among breeding grounds, indicating natal homing to breeding grounds in these species (Genner et al., 2010b). Fish in our sample were mainly caught by hook and line (by artisanal fishermen) and are therefore unlikely to comprise a large portion of breeding and fry-guarding individuals, which purportedly do not feed at all (Poll,1956; Kuwamura, 1986; Fohrman, 1994) and hence cannot be caught with bait. Consequently, our sample does not allow inferences on natal homing, as these large and mobile fish may have been collected far from their breeding grounds.

Despite the old evolutionary age of the *B. microlepis*-lineage, which dates back to the very onset of the Lake Tanganyika radiation, its mtDNA genealogy coalesces in the very recent past. The high haplotype diversity and low nucleotide diversity observed in *B. microlepis*, combined with the old age of the lineage, are typical for species that experienced population growth following a period of low effective population size (Grant & Bowen, 1998). Indeed, our data do not reject a scenario of population growth in the recent past, although the signature of expansion is not entirely compelling (Fig. 2). A possible scenario includes a decline in numbers during the East African megadrought period at ~135–75 KYA, when the lake level dropped by up to 435 m below the present level (which was still not enough to separate the three subbasins into three distinct lakes; McGlue et al., 2008) and the inhabitable lake area was reduced considerably. The subsequent rise of the lake level brought an expansion of the available habitat, which may have triggered a steady rise of *B. microlepis* stock numbers.

Additionally, large variance in reproductive success and stochasticity of juvenile mortality might have contributed to the low levels of mtDNA divergence (Hedgecock, 1994). Recent theoretical work has demonstrated that large variance in reproductive success produces shallow mitochondrial genealogies in species with high reproductive potential (e.g., Hedrick, 2005; Sargsyan & Wakeley, 2008; Hoban et al., 2013). Empirical support for this hypothesis comes from numerous studies on highly fecund taxa that showed unexpectedly shallow phylogenies and small effective population sizes (e.g., Li & Hedgecock, 1998; Arnason, 2004; Christie et al., 2010; Hedgecock & Pudovkin, 2011). *B. microlepis* is a highly fecund substrate breeding cichlid that produces up to 12,000 eggs per brood. Parents defend their offspring for at least 9 months. At that time, the offspring has grown to an average length of about 15 cm and has attained a size too big to be preyed upon by most other predators in the lake, except the large lates perches (Latidae) (Konings, 1998; Büscher,2009). The fry are certainly most vulnerable shortly after becoming mobile, when they hover above the nest in a dense cloud. Usually, predators rapidly decimate the brood to a considerable extent (Konings,1998). Large clutches and the long guarding phase predispose *B. microlepis* to considerable variance in reproductive success. Given the high mobility of this species, haplotypes of particularly successfully reproducing mothers could spread quickly and periodically replace current common ancestors.

Another puzzle, given the age of the lineage, is its failure to seed more than a single species. The other lineages of predatory benthopelagic cichlids in Lakes Malawi and Tanganyika are typically species poor as well, but nevertheless radiated into at few distinct taxa (Turner, 1996; Genner et al., 2008; Koblmüller et al.,2008). A meta-analysis of lacustrine cichlid assemblages suggested that the major factors that predispose cichlids to adaptive radiations are ecological opportunity and intrinsic lineage-specific traits related to sexual selection (Wagner et al., 2012). Compared to the benthopelagic, polygynous mouthbrooders in Lakes Tanganyika and Malawi, sexual selection has probably been weak in *B. microlepis*, judging by its monogamous mating system and lack of sexual dimorphism. Moreover, the community structure in Lake Tanganyika may also have played a significant role in preventing a radiation of the Boulengerochromini. In Lake Tanganyika, numerous piscivorous fish species from other (cichlid and non-cichlid) lineages coexist with *B. microlepis*, including the smaller piscivores in the genera *Lepidiolamprologus* and *Bathybates* as well as *Hemibates stenosoma*, and several endemic species of clariid catfish and lates perches that grow considerably larger than *B. microlepis*. Hence, available niche space of *B. microlepis*, as determined by body size, is probably restricted by heterospecific trophic competition. The extant piscivorous cichlid lineages colonized Lake Tanganyika roughly simultaneously, shortly after the establishment of deep-water conditions (Koblmüller et al.,2008; no data are available for late perches and clariid catfish). Sexual selection in the mouthbrooding piscivores and geographic isolation among the littoral substrate breeders may have given these lineages a head start in speciation, such that the Boulengerochromini ancestor quickly found the neighboring niches occupied. Contrary to restricted diversification, extinction may have eliminated traces of past speciation in the Boulengerochromini. Unfortunately, no means exist to reconstruct historic diversity beyond the coalescence of the extant mitochondrial haplotypes.

To conclude, we show that the mitochondrial genealogy of *B. microlepis* is very shallow despite the old age of the lineage and that there is no indication of phylogeographic structuring. The observed patterns of genetic diversity and divergence may be the product of recent population growth and large variance in reproductive success. Low potential for sexual selection and limited trophic niche space may have hindered speciation of the Boulengerochromini. Despite their old age, the Boulengerochromini are the only known cichlid tribe comprising only a single species, the giant cichlid from Lake Tanganyika.

## Supplementary Material

Supplementary Material

## References

[R1] Anseeuw D, Raeymaekers JAM, Busselen P, Verheyen E, Snoeks J (2011). Low genetic and morphometric intraspecific divergence in peripheral *Copadichromis* populations (Perciformes: Cichlidae) in the Lake Malawi basin. International Journal of Evolutionary Biology.

[R2] Arnason E (2004). Mitochondrial cytochrome *b* variation in the high-fecundity Atlantic cod: trans-Atlantic clines and shallow gene genealogy. Genetics.

[R3] Bandelt JJ, Forster P, Rohl A (1999). Median-joining networks for inferring intraspecific phylogenies. Molecular Biology and Evolution.

[R4] Bayona JDR (1991a). Some aspects of the biology of Kuhe, *Boulengerochromis microlepis*, in the Kigoma region, eastern coast of Lake Tanganyika. African Study Monographs.

[R5] Bayona JDR (1991b). Species composition and some observations on the littoral fishes based on beach-seining in the Kigoma region, eastern coast of Lake Tanganyika. African Study Monographs.

[R6] Büscher H (2009). Beobachtungen zum Brutverhalten von *Boulengerochromis microlepis*. DCG-Informationen.

[R7] Christie MR, Johnson DW, Stallings CD, Hixon MA (2010). Self-recruitment and sweepstakes reproduction amid extensive gene flow in a coral-reef fish. Molecular Ecology.

[R8] Cohen AS, Lezzar KE, Tiercelin JJ, Soreghan M (1997). New paleogeographic and lake-level reconstructions of Lake Tanganyika: implications for tectonic, climatic and biological evolution in a rift lake. Basin Research.

[R9] Coulter GW (1991). Lake Tanganyika and Its Life.

[R10] Crapon de Crapona M-D (1986). Are ‘preferences’ and ‘tolerances’ in cichlid mate choice important for speciation?. Journal of Fish Biology.

[R11] Drummond AJ, Rambaut A (2007). BEAST: Bayesian evolutionary analysis by sampling trees. BMC Evolutionary Biology.

[R12] Duftner N, Koblmüller S, Sturmbauer C (2005). Evolutionary relationships of the Limnochromini, a tribe of benthic deepwater cichlid fish endemic to Lake Tanganyika, East Africa. Journal of Molecular Evolution.

[R13] Duftner N, Sefc KM, Koblmüller S, Nevado B, Verheyen E, Phiri H, Sturmbauer C (2006). Distinct population structure in a phenotypically homogeneous rock-dwelling cichlid fish from Lake Tanganyika. Molecular Ecology.

[R14] Duftner N, Sefc KM, Koblmüller S, Salzburger W, Taborsky M, Sturmbauer C (2007). Parallel evolution of facial stripe patterns in the *Neolamprologus brichardi/pulcher* species complex endemic to Lake Tanganyika. Molecular Phylogenetics and Evolution.

[R15] Dunz AR, Schliewen UK (2013). Molecular phylogeny and revised classification of the haplotilapiine cichlid fishes formerly referred to as “Tilapia”. Molecular Phylogenetics and Evolution.

[R16] Dupanloup I, Schneider S, Excoffier L (2002). A simulated annealing approach to define the genetic structure of populations. Molecular Ecology.

[R17] Excoffier L, Lischer HE (2010). Arlequin suite ver 3.5: a new series of programs to perform population genetic analyses under Linux and Windows. Molecular Ecology Resources.

[R18] Fohrman K (1994). Bred in the aquarium: *Boulengerochromis microlepis*. Cichlids Yearbook.

[R19] Friedman M, Keck BP, Dornburg RI, Eytan CH, Martin CD, Hulsey PC, Wainwright, Near TJ (2013). Molecular and fossil evidence place the origin of cichlid fishes long after Gondwanan rifting. Proceedings of the Royal Society B: Biological Sciences.

[R20] Fryer G, Iles TD (1972). The Cichlid Fishes of the Great Lakes of Africa.

[R21] Fu YX (1997). Statistical tests of neutrality of mutations against population growth, hitchhiking and background selection. Genetics.

[R22] Genner MJ, Seehausen O, Lunt DH, Joyce SA, Shaw PW, Carvalho GR, Turner GF (2007). Age of cichlids: new dates for ancient lake fish radiations. Molecular Biology and Evolution.

[R23] Genner MJ, Nichols P, Shaw PW, Carvalho GR, Robinson RL, Turner GF (2008). Genetic homogeneity among breeding grounds and nursery areas of an exploited Lake Malawi cichlid fish. Freshwater Biology.

[R24] Genner MJ, Knight ME, Haeseler MP, Turner GF (2010a). Establishment and expansion of Lake Malawi rock fish populations after a dramatic Late Pleistocene lake level rise. Molecular Ecology.

[R25] Genner MJ, Nichols P, Shaw PW, Carvalho GR, Robinson RL, Turner GF (2010b). Population structure on breeding grounds of Lake Malawi’s ‘twilight zone’ cichlid fishes. Journal of Biogeography.

[R26] Grant WS, Bowen BW (1998). Shallow population histories in deep evolutionary lineages of marine fishes: insights from sardines and anchovies and lessons for conservation. Journal of Heredity.

[R27] Hedgecock D, Beaumont A (1994). Does variance in reproductive success limit effective population size of marine organisms?. Genetics and Evolution of Aquatic Organisms.

[R28] Hedgecock D, Pudovkin AI (2011). Sweepstakes reproductive success in highly fecund marine fish and shellfish: a review and commentary. Bulletin of Marine Science.

[R29] Hedrick P (2005). Large variance in reproductive success and the *N_e_*/*N* ratio. Evolution.

[R30] Hoban SM, Mezzavilla M, Gaggiotti OE, Benazzo A, van Oosterhout C, Bertorelle G (2013). High variance in reproductive success generates a false signature of a genetic bottleneck in populations of constant size: a simulation study. BMC Bioinformatics.

[R31] Kawabata M, Mihigo NYK (1982). Littoral fish fauna near Uvira, northwestern end of Lake Tanganyika. African Study Monographs.

[R32] Klett V, Meyer A (2002). What, if anything, is a Tilapia? Mitochondrial ND2 phylogeny of tilapiines and the evolution of parental care systems in the African cichlid fishes. Molecular Biology and Evolution.

[R33] Koblmüller S, Duftner N, Katongo C, Phiri H, Sturmbauer C (2005). Ancient divergence in bathypelagic Lake Tanganyika deepwater cichlids: mitochondrial phylogeny of the tribe Bathybatini. Journal of Molecular Evolution.

[R34] Koblmüller S, Sefc KM, Duftner N, Warum M, Sturmbauer C (2007). Genetic population structure as indirect measure of dispersal ability in a Lake Tanganyika cichlid. Genetica.

[R35] Koblmüller S, Sefc KM, Sturmbauer C (2008). The Lake Tanganyika cichlid species assemblage: recent advances in molecular phylogenetics. Hydrobiologia.

[R36] Koblmüller S, Duftner N, Sefc KM, Aigner U, Rogetzer M, Sturmbauer C (2009). Phylogeographic structure and gene flow in the scale-eating cichlid *Perissodus microlepis* (Teleostei, Perciformes, Cichlidae) in southern Lake Tanganyika. Zoologica Scripta.

[R37] Koblmüller S, Salzburger W, Obermüller B, Eigner E, Sturmbauer C, Sefc KM (2011). Separated by sand, fused by dropping water: habitat barriers and fluctuating water levels steer the evolution of rock-dwelling cichlid populations. Molecular Ecology.

[R38] Koch M, Koblmüller S, Sefc KM, Duftner N, Katongo C, Sturmbauer C (2007). Evolutionary history of the endemic Lake Tanganyika cichlid fish *Tylochromis polylepis*: a recent intruder to a mature adaptive radiation. Journal of Zoological Systematics and Evolutionary Research.

[R39] Konings A (1998). Tanganyika Cichlids in Their Natural Habitat.

[R40] Kuwamura T (1986). Substratum spawning and biparental guarding of the Tanganyikan cichlid *Boulengerochromis microlepis*, with notes on its life history. Physiology and Ecology Japan.

[R41] Lee W-J, Conroy J, Howell WH, Kocher TD (1995). Structure and evolution of the teleost mitochondrial control region. Journal of Molecular Evolution.

[R42] Li G, Hedgecock D (1998). Genetic heterogeneity, detected by PCR-SSCP, among samples of larval Pacific oysters (*Crassostrea gigas*) supports the hypothesis of large variance in reproductive success. Canadian Journal of Fisheries and Aquatic Sciences.

[R43] Librado P, Rozas J (2009). DnaSP v5: a software for comprehensive analysis of DNA polymorphism data. Bioinformatics.

[R44] Liem KF (1973). Evolutionary strategies and morphological innovations: cichlid pharyngeal jaws. Systematic Zoology.

[R45] Markert JA, Arnegard ME, Danley PD, Kocher TD (1999). Biogeography and population genetics of the Lake Malawi cichlid *Melanochromis auratus*: habitat transience, philopatry and speciation. Molecular Ecology.

[R46] Matthes H (1961). *Boulengerochromis microlepis*, a Lake Tanganyika fish of economic importance. Bulletin Aquatic Biology.

[R47] Mayr E (1963). Animal Species and Evolution.

[R48] McGlue MM, Lezzar KE, Cohen AS, Russell JM, Tiercelin JJ, Felton AA, Mbede E, Nkotagu HH (2008). Seismic records of late Pleistocene aridity in Lake Tanganyika, tropical East Africa. Journal of Palaeolimnology.

[R49] Meyer A, Knowles LL, Verheyen E (1996). Widespread geographic distribution of mitochondrial haplotypes in rock-dwelling cichlid fishes from Lake Tanganyika. Molecular Ecology.

[R50] Minin VN, Bloomquist EW, Suchard MA (2008). Smooth skyride through a rough sykline: Bayesian coalescent based inference of population dynamics. Molecular Biology and Evolution.

[R51] Muschick M, Indermaur A, Salzburger W (2012). Convergent evolution within an adaptive radiation of cichlid fishes. Current Biology.

[R52] Nevado B, Koblmüller S, Sturmbauer C, Snoeks J, Usano-Alemany J, Verheyen E (2009). Complete mitochondrial DNA replacement in a Lake Tanganyika cichlid fish. Molecular Ecology.

[R53] Nevado B, Mautner S, Sturmbauer C, Verheyen E (2013). Water-level fluctuations and metapopulation dynamics as drivers of genetic diversity in populations of three Tanganyikan cichlid fish species. Molecular Ecology.

[R54] Nishida M, Kawanabe H, Hori M, Nagoshi M (1997). Phylogenetic relationships and evolution of Tanganyika cichlids. Fish Communities in Lake Tanganyika.

[R55] Pereyra R, Taylor MI, Turner GF, Rico C (2004). Variation in habitat preferences and population structure among three species of the Lake Malawi cichlid genus *Protomelas*. Moelcular Ecology.

[R56] Poll M (1956). Poissons Cichlidae. Exploration hydrobiologique du Lac Tanganika (1946-1947): Résultates scientifiques.

[R57] Polzin T, Daneschmand SV (2003). On Steiner trees and minimum spanning trees in hypergraphs. Operations Research Letters.

[R58] Posada D (2008). jModelTest: phylogenetic model averaging. Molecular Biology and Evolution.

[R59] Rambaut A, Drummond AJ (2009). Tracer v1.5.

[R60] Ramos-Onsins SE, Rozas J (2002). Statistical properties of new neutrality tests against population growth. Molecular Biology and Evolution.

[R61] Rico C, Turner GF (2002). Extreme microallopatric divergence in a cichlid species from Lake Malawi. Molecular Ecology.

[R62] Salzburger W (2009). The interaction of sexually and naturally selected traits in the adaptive radiations of cichlid fishes. Molecular Ecology.

[R63] Salzburger W, Meyer A, Baric S, Verheyen E, Sturmbauer C (2002). Phylogeny of the Lake Tanganyika cichlid species flock and ist relationships to Central- and East African haplochromine cichlid fish faunas. Systematic Biology.

[R64] Sanderson MJ, Donoghue MJ (1994). Shifts in diversification rate with the origin of angiosperms. Science.

[R65] Sargsyan O, Wakeley J (2008). A coalescent process with simultaneous multiple mergers for approximating the gene genealogies of many marine organisms. Theoretical Population Biology.

[R66] Scholz CA, Johnson TC, Cohen AS, King JW, Peck JA, Overpeck JT, Talbot MR, Brown ET, Kalindafe L, Amoako PYO, Lyons RP, Shanahan TM, Castaneda IS, Heil CW, Forman SL, McHargue LR, Beuning KR, Gomez J, Pierson J (2007). East African megadroughts between 135 and 75 thousand years ago and bearing on early-modern human origins. Proceedings of the National Academy of Sciences of the United States of America.

[R67] Schwarzer J, Misof B, Tautz D, Schliewen UK (2009). The root of the East African cichlid radiations. BMC Evolutionary Biology.

[R68] Seehausen O, Witte F, Katunzi EF, Smits J, Bouton N (1997). Patterns of the remnant cichlid fauna in southern Lake Tanganyika. Conservation Biology.

[R69] Sefc KM, Baric S, Salzburger W, Sturmbauer C (2007). Species-specific population structure in rock-specialized sympatric cichlid species in Lake Tanganyika, East Africa. Journal of Molecular Evolution.

[R70] Shaw PW, Turner GF, Idid MR, Robinson RL, Carvalho GR (2000). Genetic population structure indicates sympatric speciation of Lake Malawi pelagic cichlids. Proceedings of the Royal Society London Series B: Biological Sciences.

[R71] Sturmbauer C (1998). Explosive speciation in cichlid fishes of the African Great Lakes: a dynamic model of adaptive radiation. Journal of Fish Biology.

[R72] Sturmbauer C, Baric S, Salzburger W, Rüber L, Verheyen E (2001). Lake level fluctuations synchronize genetic divergence of cichlid fishes in African lakes. Molecular Biology and Evolution.

[R73] Sturmbauer C, Fuchs C, Harb G, Damm E, Duftner N, Maderbacher M, Koch M, Koblmüller S (2008). Abundance, distribution, and territory areas of rock-dwelling Lake Tanganyika cichlid fish species. Hydrobiologia.

[R74] Sturmbauer C, Husemann M, Danley P, Zachos FE, Habel JC (2011). Explosive speciation and adaptive radiation of East African cichlid fishes. Distribution and Protection of Conservation Priority Areas.

[R75] Tajima F (1989). Statistical method for testing the neutral mutation hypothesis by DNA polymorphism. Genetics.

[R76] Takahashi T (2003). Systematics of Tanganyikan cichlid fishes (Teleostei: Perciformes). Ichthyological Research.

[R77] Takahashi T, Watanabe K, Munehara H, Rüber L, Hori M (2009). Evidence for divergent natural selection of a Lake Tanganyika cichlid inferred from repeated radiations in body size. Molecular Ecology.

[R78] Tamura K, Peterson D, Stecher G, Nei M, Kumar S (2011). MEGA5: molecular evolutionary genetics analysis using maximum likelihood, evolutionary distance, and maximum parsimony methods. Molecular Biology and Evolution.

[R79] Taylor MI, Verheyen E (2001). Microsatellite data reveals weak population substructuring in *Copadichromis* sp. “virginalis kajose”, a demersal cichlid from Lake Malawi, Africa. Journal of Fish Biology.

[R80] Taylor MI, Rüber L, Verheyen E (2001). Microsatellites reveal high levels of population substructuring in the species-poor eretmodine cichlid lineage from Lake Tanganyika. Proceedings of the Royal Society London Series B: Biological Sciences.

[R81] Turelli M, Barton NH, Coyne JA (2001). Theory and speciation. Trends in Ecology and Evolution.

[R82] Turner GF (1996). Offshore Cichlids of Lake Malawi.

[R83] Turner GF, Robinson RL, Ngatunga BP, Shaw PW, Carvalho GR, Cowx IG (2002). Pelagic cichlid fishes of Lake Malawi/Nyasa: biology, management and conservation. Management and Ecology of Lake and Reservoir Fisheries.

[R84] Van Oppen MJH, Turner GF, Rico C, Deutsch JC, Ibrahim KM, Robinson RL, Hewitt GM (1997). Unusually fine-scale genetic structuring found in rapidly speciating Malawi cichlid fishes. Proceedings of the Royal Society London Series B: Biological Sciences.

[R85] Van Steenberge M, Vanhove MPM, Breman FC, Snoeks J (2014). Complex geographical variation patterns in *Tropheus duboisi* Marlier, 1959 (Perciformes, Cichlidae) from Lake Tanganyika. Hydrobiologia.

[R86] Wagner CE, McCune AR (2009). Contrasting patterns of spatial genetic structure in sympatric rock-dwelling cichlid fishes. Evolution.

[R87] Wagner CE, Harmon LJ, Seehausen O (2012). Ecological opportunity and sexual selection together predict adaptive radiation. Nature.

[R88] Walsh PS, Metzger DW, Higuchi R (1991). Chelex 100 as a medium for simple extraction of DNA for PCR-based typing from forensic material. BioTechniques.

[R89] Wright S (1978). Evolution and the Genetic of Populations 4. Variability Within and Among Natural Populations.

