# Supplementary material for: Big fish, little divergence: phylogeography of Lake Tanganyika’s giant cichlid, Boulengerochromis microlepis
Source: Hydrobiologia. Author manuscript; Available in PMC 2015 May 14. (PMC4430823; doi:10.1007/s10750-014-1863-z)
Supplement: Supplementary Material [file NIHMS59145-supplement-suppl_1.pdf]

**Supplementary Table 1** Sample IDs, sampling localities plus coordinates, sampling years and haplotypes of *Boulenegerochromis microlepis*.

| Sample ID | Locality | Coordinates | Year | Haplotype |
|---|---|---|---|---|
| 5977 | Mpulungu fishmarket | 8°46’ S; 31°07’ E | 2006 | Ht24 |
| 6113 | Kalambo Lodge | 8°37’ S; 31°37’ E | 2006 | Ht14 |
| 6121 | Mpulungu fishmarket | 8°46’ S; 31°07’ E | 2004 | Ht22 |
| 6122 | Mpulungu fishmarket | 8°46’ S; 31°07’ E | 2004 | Ht14 |
| 6123 | Mpulungu fishmarket | 8°46’ S; 31°07’ E | 2004 | Ht14 |
| 6127 | Mpulungu fishmarket | 8°46’ S; 31°07’ E | 2004 | Ht14 |
| 6132 | Wonzye | 8°42’ S; 31°08’ E | 2004 | Ht14 |
| 6135 | Mpulungu fishmarket | 8°46’ S; 31°07’ E | 2003 | Ht20 |
| 6136 | Mpulungu fishmarket | 8°46’ S; 31°07’ E | 2003 | Ht22 |
| 6137 | Mpulungu fishmarket | 8°46’ S; 31°07’ E | 2003 | Ht22 |
| 6157 | Mpulungu | 8°46’ S; 31°07’ E | 2001 | Ht14 |
| 7692 | Mkangasi – upstream Luega River | 6°39’ S; 30°21’ E | 2007 | Ht28 |
| 7693 | Mkangasi – upstream Luega River | 6°39’ S; 30°21’ E | 2007 | Ht29 |
| 13454 | Mpulungu fishmarket | 8°46’ S; 31°07’ E | 2004 | Ht13 |
| 13455 | Mpulungu fishmarket | 8°46’ S; 31°07’ E | 2004 | Ht22 |
| 14275 | Mpulungu fishmarket | 8°46’ S; 31°07’ E | 2003 | Ht14 |
| 14277 | Mpulungu fishmarket | 8°46’ S; 31°07’ E | 2003 | Ht10 |
| 14279 | Mpulungu fishmarket | 8°46’ S; 31°07’ E | 2003 | Ht22 |
| 14280 | Mpulungu fishmarket | 8°46’ S; 31°07’ E | 2003 | Ht14 |
| 14281 | Mpulungu fishmarket | 8°46’ S; 31°07’ E | 2003 | Ht20 |
| 14282 | Mpulungu fishmarket | 8°46’ S; 31°07’ E | 2003 | Ht20 |
| 14283 | Mpulungu fishmarket | 8°46’ S; 31°07’ E | 2003 | Ht25 |
| 14284 | Mpulungu fishmarket | 8°46’ S; 31°07’ E | 2003 | Ht14 |
| 14285 | Mpulungu fishmarket | 8°46’ S; 31°07’ E | 2003 | Ht18 |
| 14286 | Mpulungu fishmarket | 8°46’ S; 31°07’ E | 2003 | Ht14 |
| 14287 | Mpulungu fishmarket | 8°46’ S; 31°07’ E | 2003 | Ht14 |
| 14288 | Mpulungu fishmarket | 8°46’ S; 31°07’ E | 2003 | Ht1 |
| 14289 | Mpulungu fishmarket | 8°46’ S; 31°07’ E | 2003 | Ht14 |
| 14290 | Mpulungu fishmarket | 8°46’ S; 31°07’ E | 2003 | Ht14 |
| 14291 | Mpulungu fishmarket | 8°46’ S; 31°07’ E | 2003 | Ht14 |
| 14292 | Mpulungu fishmarket | 8°46’ S; 31°07’ E | 2003 | Ht1 |
| 14294 | Mpulungu fishmarket | 8°46’ S; 31°07’ E | 2007 | Ht22 |
| 14296 | Mpulungu fishmarket | 8°46’ S; 31°07’ E | 2007 | Ht22 |
| 14298 | Mpulungu fishmarket | 8°46’ S; 31°07’ E | 2007 | Ht18 |
| 14299 | Mpulungu fishmarket | 8°46’ S; 31°07’ E | 2007 | Ht14 |
| 14300 | Kipili | 7°27’ S; 30°35’ E | 2007 | Ht14 |
| 14301 | N of Mabilibili | 6°26’ S; 29°54’ E | 2007 | Ht4 |
| 14302 | N of Mabilibili | 6°26’ S; 29°54’ E | 2007 | Ht7 |
| 14303 | Mpulungu fishmarket | 8°46’ S; 31°07’ E | 2012 | Ht24 |
| 14304 | Mpulungu fishmarket | 8°46’ S; 31°07’ E | 2012 | Ht22 |
| 14305 | Mpulungu fishmarket | 8°46’ S; 31°07’ E | 2003 | Ht25 |
| 14306 | Mpulungu fishmarket | 8°46’ S; 31°07’ E | 2012 | Ht14 |
| 14307 | Mpulungu fishmarket | 8°46’ S; 31°07’ E | 2012 | Ht1 |
| 14308 | Kalambo Lodge | 8°37’ S; 31°37’ E | 2012 | Ht20 |
| 14309 | Kalambo Lodge | 8°37’ S; 31°37’ E | 2012 | Ht6 |
| 14310 | Mpulungu fishmarket | 8°46’ S; 31°07’ E | 2012 | Ht14 |
| 14311 | Mpulungu fishmarket | 8°46’ S; 31°07’ E | 2012 | Ht14 |
| 14312 | Kalambo Lodge | 8°37’ S; 31°37’ E | 2012 | Ht1 |

| | | | | |
|---|---|---|---|---|
| 14313 | Bujumbura fishmarket | 3°23’ S; 29°21’ E | 2013 | Ht12 |
| 14314 | Bujumbura fishmarket | 3°23’ S; 29°21’ E | 2013 | Ht2 |
| 14315 | Bujumbura fishmarket | 3°23’ S; 29°21’ E | 2013 | Ht27 |
| 14316 | Bujumbura fishmarket | 3°23’ S; 29°21’ E | 2013 | Ht26 |
| 14317 | Bujumbura fishmarket | 3°23’ S; 29°21’ E | 2013 | Ht26 |
| 14320 | Mpulungu fishmarket | 8°46’ S; 31°07’ E | 2001 | Ht1 |
| 14322 | Mpulungu fishmarket | 8°46’ S; 31°07’ E | 2003 | Ht21 |
| 14323 | Mpulungu fishmarket | 8°46’ S; 31°07’ E | 2003 | Ht22 |
| 14324 | Mpulungu fishmarket | 8°46’ S; 31°07’ E | 2003 | Ht22 |
| 14325 | Mpulungu fishmarket | 8°46’ S; 31°07’ E | 2003 | Ht22 |
| 14326 | Mpulungu fishmarket | 8°46’ S; 31°07’ E | 2003 | Ht14 |
| 14327 | Mpulungu fishmarket | 8°46’ S; 31°07’ E | 2003 | Ht18 |
| 14331 | Kalambo Lodge | 8°37’ S; 31°37’ E | 2006 | Ht14 |
| 14334 | Chisanze | 8°31’ S; 30°32’ E | 2006 | Ht5 |
| 14335 | Chimba | 8°25’ S; 30°27’ E | 2006 | Ht19 |
| 14336 | Mpulungu fishmarket | 8°46’ S; 31°07’ E | 2006 | Ht22 |
| 14337 | Mpulungu fishmarket | 8°46’ S; 31°07’ E | 2006 | Ht17 |
| 14338 | Mpulungu fishmarket | 8°46’ S; 31°07’ E | 2006 | Ht22 |
| 14339 | Kalambo Lodge | 8°37’ S; 31°37’ E | 2006 | Ht14 |
| 14340 | Kalambo Lodge | 8°37’ S; 31°37’ E | 2006 | Ht22 |
| 14341 | Kalambo Lodge | 8°37’ S; 31°37’ E | 2006 | Ht14 |
| 14342 | Kalambo Lodge | 8°37’ S; 31°37’ E | 2006 | Ht25 |
| 14343 | Kalambo Lodge | 8°37’ S; 31°37’ E | 2006 | Ht14 |
| 14344 | Kalambo Lodge | 8°37’ S; 31°37’ E | 2006 | Ht14 |
| 14345 | Kalambo Lodge | 8°37’ S; 31°37’ E | 2006 | Ht14 |
| 14346 | Katete | 8°20’ S; 30°30’ E | 2006 | Ht23 |
| 14349 | Mpulungu fishmarket | 8°46’ S; 31°07’ E | 2004 | Ht1 |
| 14350 | Mpulungu fishmarket | 8°46’ S; 31°07’ E | 2004 | Ht14 |
| 14353 | Mpulungu fishmarket | 8°46’ S; 31°07’ E | 2007 | Ht18 |
| 14355 | Mpulungu fishmarket | 8°46’ S; 31°07’ E | 2007 | Ht21 |
| 14356 | Kipili | 7°27’ S; 30°35’ E | 2007 | Ht15 |
| 14357 | Kipili | 7°27’ S; 30°35’ E | 2007 | Ht16 |
| 14358 | S of Isonga | 6°29’ S; 30°10’ E | 2007 | Ht28 |
| 14359 | N of Mabilibili | 6°26’ S; 29°54’ E | 2007 | Ht22 |
| 14361 | Nkondwe Island | 7°23’ S; 30°33’ E | 2007 | Ht3 |
| 14362 | Mulembwe | 6°07’ S; 29°16’ E | 2010 | Ht9 |
| 14363 | Mugayo | 6°47’ S; 29°34’ E | 2010 | Ht11 |
| 14364 | Moba | 7°03’ S; 29°47’ E | 2010 | Ht8 |
| 14365 | Mulembwe | 6°07’ S; 29°16’ E | 2010 | Ht9 |
| 14366 | Bujumbura fishmarket | 3°23’ S; 29°21’ E | 2013 | Ht19 |

Note that samples obtained at the fishmarkets in Mpulungu and Bujumbura might actually come from anywhere nearby these cities.

**Supplementary Table 2** GenBank accession numbers for haplotypes of *Boulengerochromis microlepis*.

| Haplotype | GenBank accession number |
|---|---|
| Ht1 | |
| Ht2 | |
| Ht3 | |
| Ht4 | |
| Ht5 | |
| Ht6 | |
| Ht7 | |
| Ht8 | |
| Ht9 | |
| Ht10 | |
| Ht11 | |
| Ht12 | |
| Ht13 | |
| Ht14 | |
| Ht15 | |
| Ht16 | |
| Ht17 | |
| Ht18 | |
| Ht19 | |
| Ht20 | |
| Ht21 | |
| Ht22 | |
| Ht23 | |
| Ht24 | |
| Ht25 | |
| Ht26 | |
| Ht27 | |
| Ht28 | |
| Ht29 | |

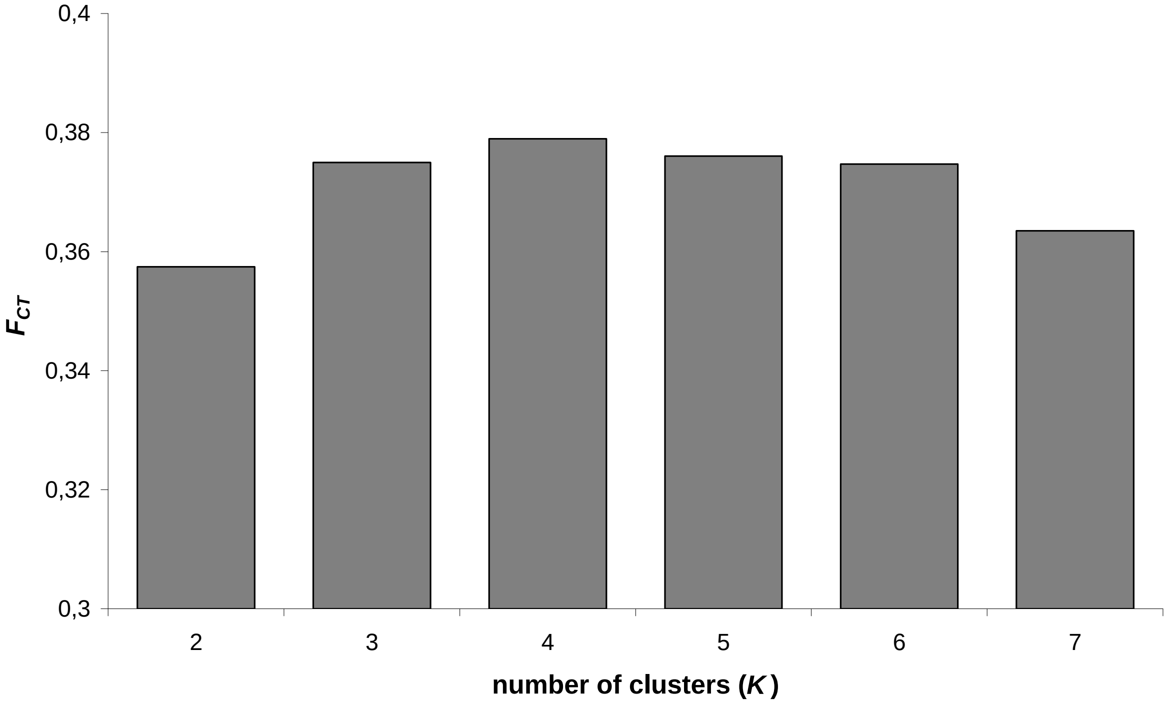


**Supplementary Fig. 1** Results of the spatial analysis of molecular variance. A maximum $F_{CT}$ was observed for $K$ = 4 (cluster I, Chisanze; cluster II, S of Isonga plus Mkangasi; cluster III, Nkondwe Island; cluster IV, the remaining locations; see Fig. 1).
